# Modeling congenital cataract in vitro using patient-specific induced pluripotent stem cells

**DOI:** 10.1038/s41536-021-00171-x

**Published:** 2021-10-01

**Authors:** Danni Lyu, Lifang Zhang, Zhenwei Qin, Shuang Ni, Jiayong Li, Bing Lu, Shengjie Hao, Qiaomei Tang, Houfa Yin, Zhijian Chen, Yong-Bin Yan, Junfeng Ji, Jiliang He, Andras Nagy, Qiuli Fu, Ke Yao

**Affiliations:** 1 Eye Center of the 2nd Affiliated Hospital, School of Medicine, Zhejiang University, Zhejiang Provincial Key Lab of Ophthalmology, Hangzhou, 310009 Zhejiang Province China; 2 Department of Environmental and Occupational Health, Zhejiang Provincial Center for Disease Control and Prevention, Hangzhou, 310051 Zhejiang Province China; 3 State Key Laboratory of Membrane Biology, School of Life Sciences, Tsinghua University, Beijing, 100084 China; 4 Center of Stem Cell and Regenerative Medicine, School of Medicine, Zhejiang University, Zhejiang Provincial Key Laboratory of Tissue Engineering and Regenerative Medicine, Hangzhou, 310058 Zhejiang Province China; 5 Institute of Environmental Medicine, School of Medicine, Zhejiang University, Hangzhou, 310058 Zhejiang Province China; 6 Lunenfeld-Tanenbaum Research Institute, Sinai Health System, and Institute of Medical Science, University of Toronto, Toronto, M5T 3H7 Ontario Canada

**Keywords:** Lens diseases, Experimental models of disease, Stem-cell research

## Abstract

Congenital cataracts are the leading cause of childhood blindness. To date, surgical removal of cataracts is the only established treatment, but surgery is associated with multiple complications, which often lead to visual impairment. Therefore, mechanistic studies and drug-candidate screening have been intrigued by the aims of developing novel therapeutic strategies. However, these studies have been hampered by a lack of an appropriate human-disease model of congenital cataracts. Herein, we report the establishment of a human congenital cataract in vitro model through differentiation of patient-specific induced pluripotent stem cells (iPSCs) into regenerated lenses. The regenerated lenses derived from patient-specific iPSCs with known causative mutations of congenital cataracts (*CRYBB2* [p. P24T] and *CRYGD* [p. Q155X]) showed obvious opacification that closely resembled that seen in patients’ cataracts in terms of opacification severity and disease course accordingly, as compared with lentoid bodies (LBs) derived from healthy individuals. Increased protein aggregation and decreased protein solubility corresponding to the patients’ cataract severity were observed in the patient-specific LBs and were attenuated by lanosterol treatment. Taken together, the in vitro model described herein, which recapitulates patient-specific clinical manifestations of congenital cataracts and protein aggregation in patient-specific LBs, provides a robust system for research on the pathological mechanisms of cataracts and screening of drug candidates for cataract treatment.

## Introduction

Human-induced pluripotent stem cells (iPSCs) provide a promising resource to establish human-disease models with individual-specific genetic backgrounds for use in pathological, pharmaceutical, and regeneration studies. Models of various diseases, such as cardiac arrhythmias, cardiomyopathy, inherited metabolic liver disorders, diabetes, and Alzheimer’s disease, have been developed based on the differentiation of patient-specific iPSCs into various cells, including cardiac myocytes, hepatocytes, pancreatic beta-cells, and neurons or tissues^1–5^. These disease models simulate patient-specific phenotypes, thus having wide applications in the study of pathological mechanisms and the screening of drug candidates under the patient-specific genetic backgrounds. However, thus far, for various reasons, including a lack of well-established protocols to regenerate a lens in vitro, no appropriate patient-specific cataract model based on iPSCs has been reported.

For decades, many efforts have been made to regenerate lenses or lentoid bodies (LBs) and lens cells in vitro from embryonic stem cells or iPSCs in vitro^6–12^. Despite the expression of lens-specific markers at specific stages of differentiation, as well as similar chemical and biological characteristics to those of a human lens, these LBs have several limitations in terms of their morphology, size, structure, and optic function, which restrict further applications. In our previous work, we made several modifications to a previously established protocol of lens regeneration in vitro, the “three-stage” protocol^12^, and established a “fried egg” method to generate LBs from human urinary cell-derived iPSCs (UiPSCs)^13^. The LBs generated using our method not only expressed various human lens-specific markers during each stage of differentiation, but also exhibited a lens-like structure and optic properties that resemble to a large extent those seen in human-lens development in morphological, molecular, and structural aspects. Recent studies confirmed highly overlapping transcriptomic and proteomic profiles of LBs derived from human iPSCs via the “fried egg” method and murine lens epithelial and fiber cells^14,15^. In addition, these studies observed closely packed lens epithelial and differentiating fiber-like cells in iPSC-derived LBs, which showed a similar ultrastructure to that of the lens^14,15^. Therefore, the availability of LBs provides an opportunity to generate cataract-disease models from individual-specific iPSCs.

A cataract is defined as opacification of the lens that leads to light scatter and visual impairment^16^. Congenital cataracts are a leading cause of childhood blindness, accounting for over one-third of all cases^17^, among which 8.3–25% of cases are inherited^18–20^. To date, nearly 40 genes have been linked to congenital cataracts^21^. As the only treatment option for congenital cataracts, surgery is considered to be of great difficulty, due to the high incidence of intraoperative and postoperative complications, severe inflammation, amblyopia, etc. Investigations of the pathological mechanisms of congenital cataracts are urgently needed to aid the development of novel therapeutic strategies.

Thus far, traditional cellular models and animal models have been utilized in mechanistic studies and drug-candidate evaluation. However, cellular models are two-dimensional and cannot fully represent the human lens with regard to its morphological, structural, and biochemical characteristics. Likewise, animal models are problematic in that they involve heterogeneity of species and genetic backgrounds. An appropriate human-disease model of congenital cataracts that recapitulates the patient’s clinical manifestation and disease course is urgently needed. In our previous study, transparent LBs derived from iPSCs of healthy individuals became opacified along with the prolonged culture duration, and opacification was further aggravated by hydrogen-peroxide treatment^22^. This human-lens aging model closely resembled the pathological process of age-related cataracts in vitro and pointed the feasibility of establishing a human-disease model of congenital cataracts using patient-specific iPSCs.

Cataracts are characterized by protein aggregation^16^. Thus, eliminating or reversing protein aggregation is considered a promising strategy for cataract treatment. Based on this idea, researchers showed that lanosterol, an intrinsic amphipathic molecule, as well as 25-hydroxycholesterol, reversed protein aggregation in lens epithelial cells in vitro and partially restored lens transparency in murine and canine models in vivo^23,24^. However, the efficacy of these compounds in an opacified human lens has not been demonstrated, thus restricting their potential clinical application. Therefore, it is urgent to establish a human-disease model that represents the characteristics of the human lens and displays similar pathological changes to those seen in human congenital cataracts. Such a model must also be able to be used to simulate the response of the human lens toward drug candidates. The “fried egg” method of LB differentiation might form the basis for a human model of cataracts. If patient-specific LBs that resemble patients’ lens characteristics could be obtained from congenital cataract patients with genetic mutations, this might provide a promising alternative model for the screening and evaluation of drug candidates to treat cataracts.

In this study, using patient-specific LBs from the UiPSCs, we attempted to establish a patient-specific congenital cataract model in vitro that recapitulated patients’ clinical manifestations, disease courses, and pathological changes. LBs from individuals in two families with the most commonly reported causative mutations of the principal soluble structural proteins in lens, *CRYBB2* (p. Q155X) and *CRYGD* (p. P24T), were included in this study. This study may provide a promising patient-specific in vitro platform for mechanistic studies of congenital cataracts and therapeutic studies of drug candidates to treat the disease.

## Results

### Clinical data of congenital cataract patients

Crystallins, consisting of α- and βγ-crystallin families, make up approximately 90% of water-soluble proteins of the mammalian lens. These highly organized proteins provide a refractive-index gradient, which allows for transparency of the lens^25^. Decreased solubility of crystallins due to various factors, including gene mutations and oxidation stress, results in lens opacification (i.e., cataracts).

**Fig. 1:**
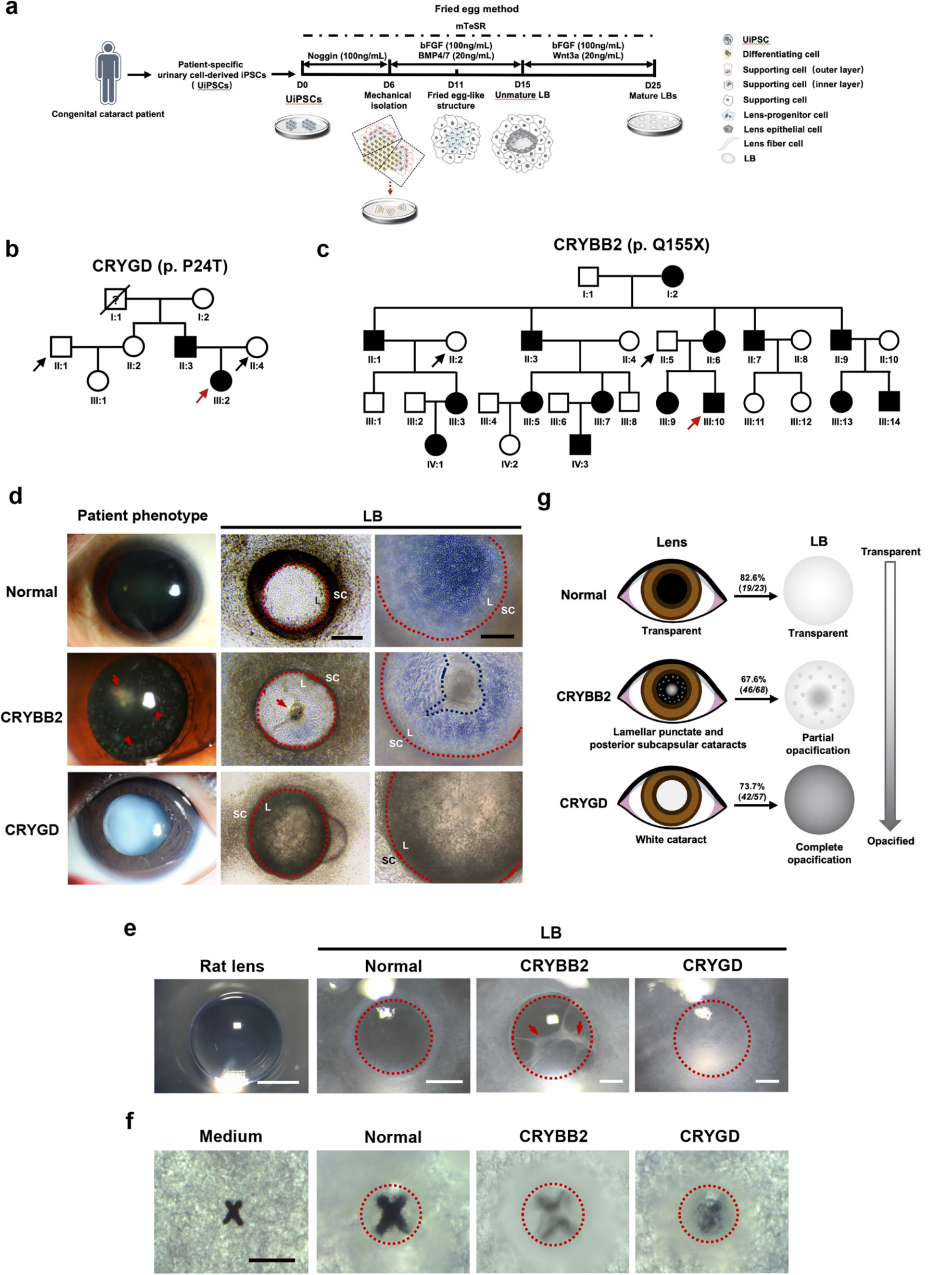
Generation of UiPSC-derived LBs from patients with congenital cataracts. **a** Schematic diagram of the generation of patient-specific LBs. **b** & **c** Pedigrees of family 1 (**b**) and family 2 (**c**). The squares and circles indicate male and female family members, respectively. The solid and open symbols represent affected and unaffected individuals, respectively. The probands are marked with red arrows. The black arrows represent the family members whose samples were collected as controls. **d** A comparison between patients’ cataract phenotypes and the morphology of mature patient-specific LBs. Left panels: Slit-lamp photographs of the healthy control, the proband (III:2) from family 1 (*CRYBB2*-mutated), and the proband (III:10) from family 2 (*CRYGD*-mutated). The red arrow indicates posterior subcapsular opacity. The red arrowheads indicate lamellar punctate opacity. Middle and right panels: Representative images of normal and patient-specific LBs on D25. The red dashed lines indicate the borders of the LBs. The red arrow and blue dashed line indicate the cord-like structure in the *CRYBB2-*mutated LBs. Written consent was obtained for publication of the photographs of the patients and the healthy control. L: LB; SC: supporting cells. Scale bars: 250 μm (middle panels), 100 μm (right panels). **e** Morphology of mature LBs derived from the normal, *CRYGD*-, and *CRYBB2*-mutated UiPSCs on D25, as well as rat lens under a dissecting microscope. The red dashed lines indicate the borders of the LBs. The red arrows indicate a punctate or cord-like opacification in a *CRYBB2*-mutated LB. Scale bars: rat lens: 2 mm; LBs: 0.5 mm. **f** The optic properties of the normal and patient-specific LBs on D25 via “X” assay under a dissecting microscope. The red dashed lines indicate the borders of the LBs. Scale bar: 1 mm. **g** Schematic of the consistence between the cataract phenotypes of the patients and the morphologies of patient-specific LBs. The ratio indicates the fraction of LBs that shared a similar morphology with the corresponding patient out of all patient-specific LBs within each group.

In this study, two congenital cataract families with distinct clinical manifestations were enrolled for collection of urine samples. Patient-specific LBs were generated from the urinary cell-derived iPSCs of patients with different mutations using the “fried egg” method (schematized in Fig. 1a). Family 1 was a three-generation Chinese family with congenital cataracts caused by the p. P24T mutation in γD-crystallin (*CRYGD*) (Fig. 1b). The proband (III:2, proband 1) was born with leukocoria in both eyes and diagnosed with bilateral white congenital cataracts under slit-lamp examination during the first visit at the age of two years (Fig. 1d, lower-left panel). Proband 1 underwent cataract surgery soon due to her poor visual acuity. Family 2 was a four-generation Chinese family with mild congenital cataracts caused by the p. Q155X mutation in βB2-crystallin (*CRYBB2*) (Fig. 1c). A slit-lamp examination showed bilateral lamellar punctate and posterior subcapsular cataracts in the proband (III:10, proband 2) during the first visit at the age of 11 years (Fig. 1d, middle-left panel). Regular follow-ups showed slow progression of the cataract, with a limited influence on the patient’s visual acuity. Thus, no further surgery was planned in the short term.

### Generation of UiPSCs from patients with congenital cataracts

Urine samples were collected from the proband and two unaffected family members from each of the two families, respectively (Fig. 1b and c). Exfoliated urinary cells collected from the urine samples were cultured to passages 1–3 and then infected with the four Yamanaka factors and induced into iPSCs as previously reported^13^. At least three independent colonies of iPSCs from each participant were selected. The phenotypes were consistent among the independent clones derived from the same participant. The expression of a set of pluripotent stem cell markers (SOX2, Nanog, SSEA4, and Tra1–81) was confirmed by an immunofluorescence assay (Supplementary Fig. 1a and b). The UiPSCs were alkaline phosphatase (AP)-positive and able to form embryonic bodies (EBs) (Supplementary Fig. 1c and d). A teratoma assay revealed tissues representative of all three germ layers, which further verified the pluripotency of the UiPSCs (Supplementary Fig. 1e). Sanger sequencing of *CRYGD* obtained from the UiPSCs derived from proband 1 confirmed a heterozygous missense mutation in *CRYGD* exon 2, at position 70 (c. 70 C > A) and the substitution of proline for threonine at position 24 (p. P24T) (Supplementary Fig. 1f). Sequence analysis of *CRYBB2* from the UiPSCs derived from proband 2 revealed a heterozygous nonsense mutation in *CRYBB2* exon 6 at position 463 (c. 463 C > T), resulting in the substitution of glutamine for a stop codon (p. Q155X) (Supplementary Fig. 1g).

### Patient-specific LBs displayed opacification similar to that seen in patients’ cataracts

Cataract is defined as lens opacification. In our previous study, mature LBs with a morphology similar to that of a normal lens (i.e., round and transparent) were obtained from healthy individual-derived UiPSCs on day 25 (D25) of differentiation (Fig. 1d)^13^. However, aberrant morphology was observed in the LBs differentiated from the *CRYGD*- and *CRYBB2*-mutated UiPSCs (named *CRYGD*-mutated LBs or *CRYBB2*-mutated LBs). On D25, the *CRYGD*-mutated LBs showed almost complete opacification, which was similar to that observed in the patient’s cataract (Fig. 1d and e). As shown by an “X” assay of the optic properties, an “X” was clearly visible under the normal LBs with magnification, whereas the “X” under the *CRYGD*-mutated LBs was severely blurred due to a significant reduction in LB translucency (Fig. 1f). These results indicated similar opacification type and severity between the *CRYGD*-mutated LBs and the cataract in proband 1, whose visual acuity was very poor at birth. Most of the *CRYBB2*-mutated LBs showed mild-to-moderate punctate or cord-like opacification, which was far milder than that in the *CRYGD*-mutated LBs. In addition, the opacification was in some ways similar to the cataract morphology in proband 2, whose visual acuity was only slightly affected and where surgery was not indicated at present (Fig. 1d and e). Optic property analysis showed a vague image of an “X” under the *CRYBB2*-mutated LBs (Fig. 1f). These results implied that the extent and type of opacification of the patient-specific LBs were largely correspondent with the patients’ cataracts (schematized in Fig. 1g).

**Fig. 2:**
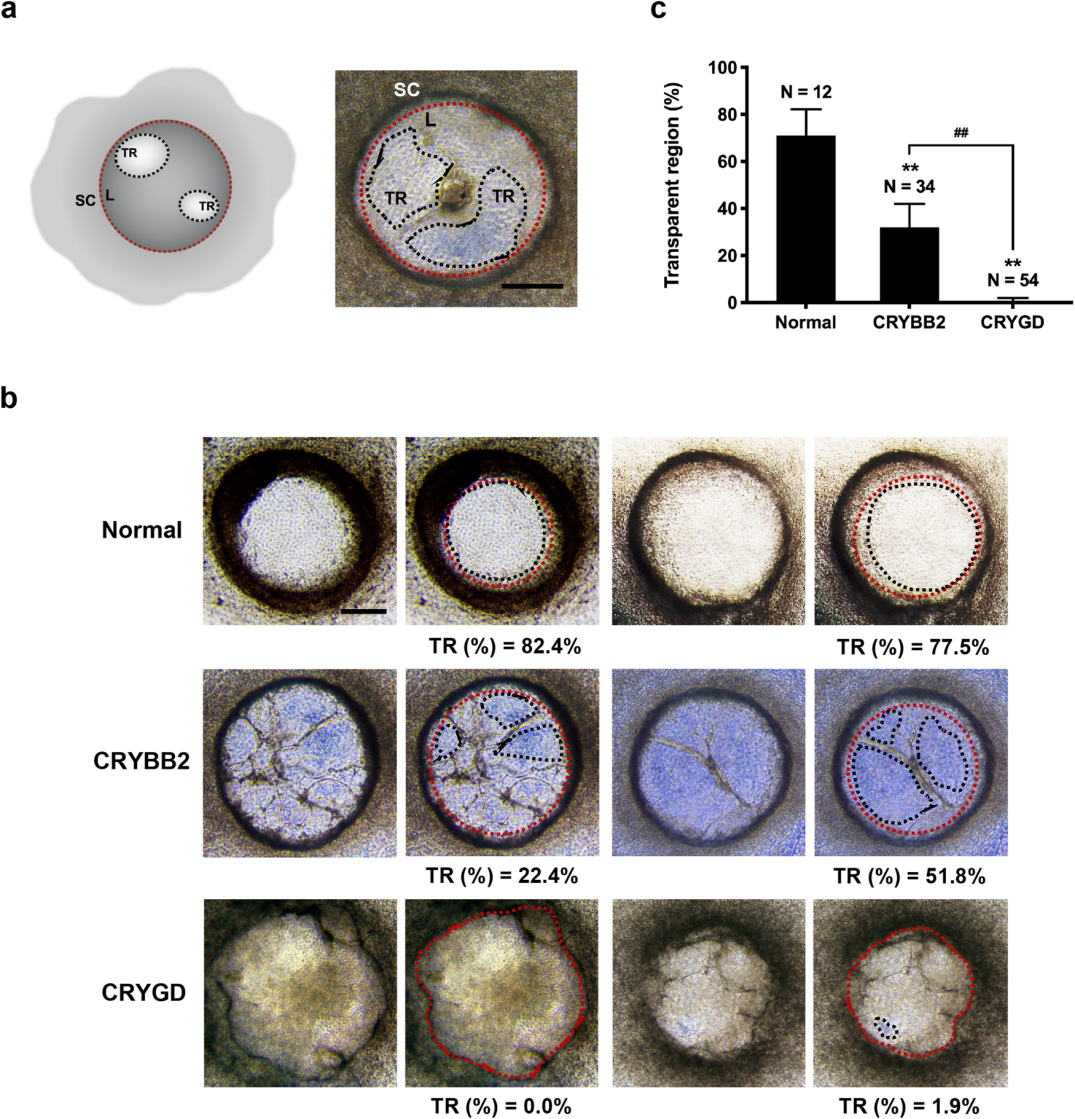
Quantitative analysis of LB opacification. **a** Left panel: Schematic illustrating the method of measuring the fractional area of transparent regions (TR%) in the LBs. Right panel: Representative image of a LB showing the method of fractional area measurement. L: LB; TR: transparent region; SC: supporting cells. The dashed red lines indicate the borders of the LBs. The blue dashed lines indicate the borders of transparent regions. Scale bars: 200 μm. **b** Representative images and the TR% of the normal, *CRYBB2*-, and *CRYGD*-mutated LBs. The dashed red lines indicate the borders of LBs. The blue dashed lines indicate the borders of transparent regions. Scale bars: 200 μm. **c** TR% analysis of the normal, *CRYBB2*-, and *CRYGD*-mutated LBs. The data are presented as mean ± SD. **p < 0.01 versus normal, ^##^*p* < 0.01 versus the indicated group.

We further quantified the degree of opacification of LBs by measuring the fractional area of transparent regions (TR%) as illustrated in Fig. 2a. A total of 12 normal LBs, 34 *CRYBB2*-mutated LBs, and 54 *CRYGD*-mutated LBs were evaluated by three ophthalmologists independently using a blinded method. Representative images of the LBs from each group and the corresponding TR% are shown in Fig. 2b. The results revealed that the TR% values of the *CRYGD*-mutated LBs (0.57 ± 1.35%) and the *CRYBB2*-mutated LBs (34.76 ± 10.85%) were significantly smaller than those of the normal LBs (84.34 ± 7.00%) (*p* < 0.01) (Fig. 2c). The TR% of the *CRYGD*-mutated LBs was also significantly smaller than that of the *CRYBB2*-mutated LBs (*p* < 0.01), indicating more severe opacification in the *CRYGD*-mutated LBs than in the *CRYBB2*-mutated LBs (Fig. 2c). The cataract in proband 1 with the *CRYGD* (p. P24T) mutation was more severe than that in proband 2 with the *CRYBB2* (p. Q155X) mutation. Thus, the TR% of the patient-specific LBs was largely consistent with the severity of the patients’ cataracts.

### Patient-specific LBs expressed mature lens-specific markers but showed protein aggregation

**Fig. 3:**
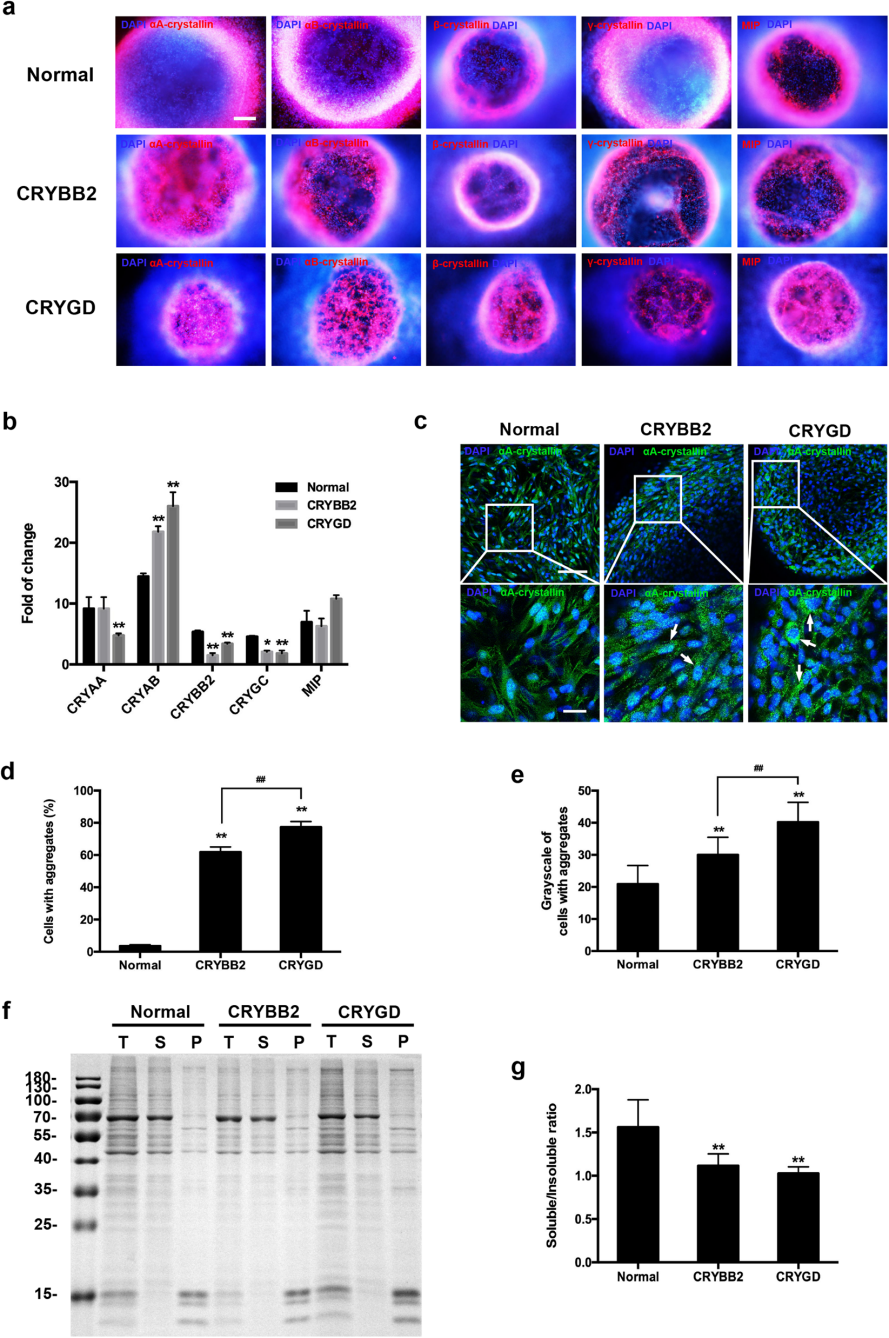
Expression characteristics of the *CRYGD*- and *CRYBB2*-mutated LBs. **a** Immunofluorescence analysis of the human mature lens-specific markers on D25. Scale bar: 200 μm. **b** qRT-PCR analysis of the human mature lens-specific markers on D25. *p < 0.05, **p < 0.01 versus normal. **c** Representative images of αA-crystallin aggregates in the *CRYGD*- and *CRYBB2*-mutated LBs on D25. Cells with inhomogeneous punctate staining (i.e., aggregates) are indicated by white arrows. Scale bars: 100 μm (upper panels), 25 μm (lower panels). **d**, **e** Quantitative analysis of the percentage of cells with protein aggregates (**d**) and the mean grayscales of the cells with aggregates (**e**) in the normal and the patient-specific LBs. At least three fields (250×) from each group were randomly chosen for analysis. **p < 0.01 versus normal, ^##^*p* < 0.01 *CRYBB2* versus *CRYGD*. **f** SDS-PAGE gel electrophoresis of the soluble and insoluble proportions of the protein of the normal and patient-specific LBs on D25. The gels derived from the same experiment and were processed in parallel. T total protein, S soluble protein, P insoluble protein. **g** Quantitative analysis of the ratio of soluble and insoluble protein. **p < 0.01 versus normal. The data are presented as the mean ± SD of experiments from at least three parallel samples per group.

To clarify the expression characteristics of the *CRYGD*- and *CRYBB2*-mutated LBs, the mature lens-specific markers *α*A-, *α*B-, *β*-, *γ*-crystallin, and the major intrinsic protein (MIP) were determined by immunofluorescence and quantitative real-time polymerase chain reaction (qRT-PCR) assays. The results revealed positive expression of *α*A-, *α*B-, *β*-, *γ*-crystallin, and MIP on D25 in the normal and the patient-specific LBs (Fig. 3a and b). Interestingly, the expression of *β*- and *γ*-crystallin in both the *CRYGD*- and *CRYBB2*-mutated LBs was significantly lower than that in the normal LBs, whereas the expression of *α*B-crystallin was significantly higher than that in the normal LBs (Fig. 3b). The possible reasons for this are discussed in the “Discussion” section.

The key pathological mechanism of cataracts is protein misfolding in the lens, leading to protein aggregation and precipitation^16^. Interestingly, the immunofluorescence assay of αA-crystallin revealed abundant puncta in the *CRYGD*- and *CRYBB2*-mutated LBs on D25, suggesting formation of protein aggregates, whereas most of the normal LBs displayed homogeneous signals (Fig. 3c). Denser puncta were observed in the *CRYGD*-mutated LBs as compared with the *CRYBB2*-mutated LBs, which may explain the more severe opacification in the former. These results were confirmed by quantitative analyses in which the percentage of the cells with aggregates was calculated and the mean grayscale of LBs was measured (Fig. 3d and e, *p* < 0.01). In addition, the soluble and insoluble proportions of the protein in the LBs were extracted and analyzed. The results of sodium dodecyl sulfate polyacrylamide gel electrophoresis (SDS-PAGE) showed that the soluble/insoluble protein ratio was significantly lower in the mature *CRYGD*- and *CRYBB2*-mutated LBs, indicating a distinct decrease in the protein solubility compared with the normal LBs (Fig. 3f, g; *p* < 0.01). The soluble/insoluble protein ratio in the *CRYBB2*-mutated LBs was slightly higher than that in the *CRYGD*-mutated LBs (*p* > 0.05). These results suggested that the opacification in the patient-specific LBs shared similar pathological mechanisms with those of cataracts.

### Patient-specific LBs revealed a slightly disordered structure

**Fig. 4:**
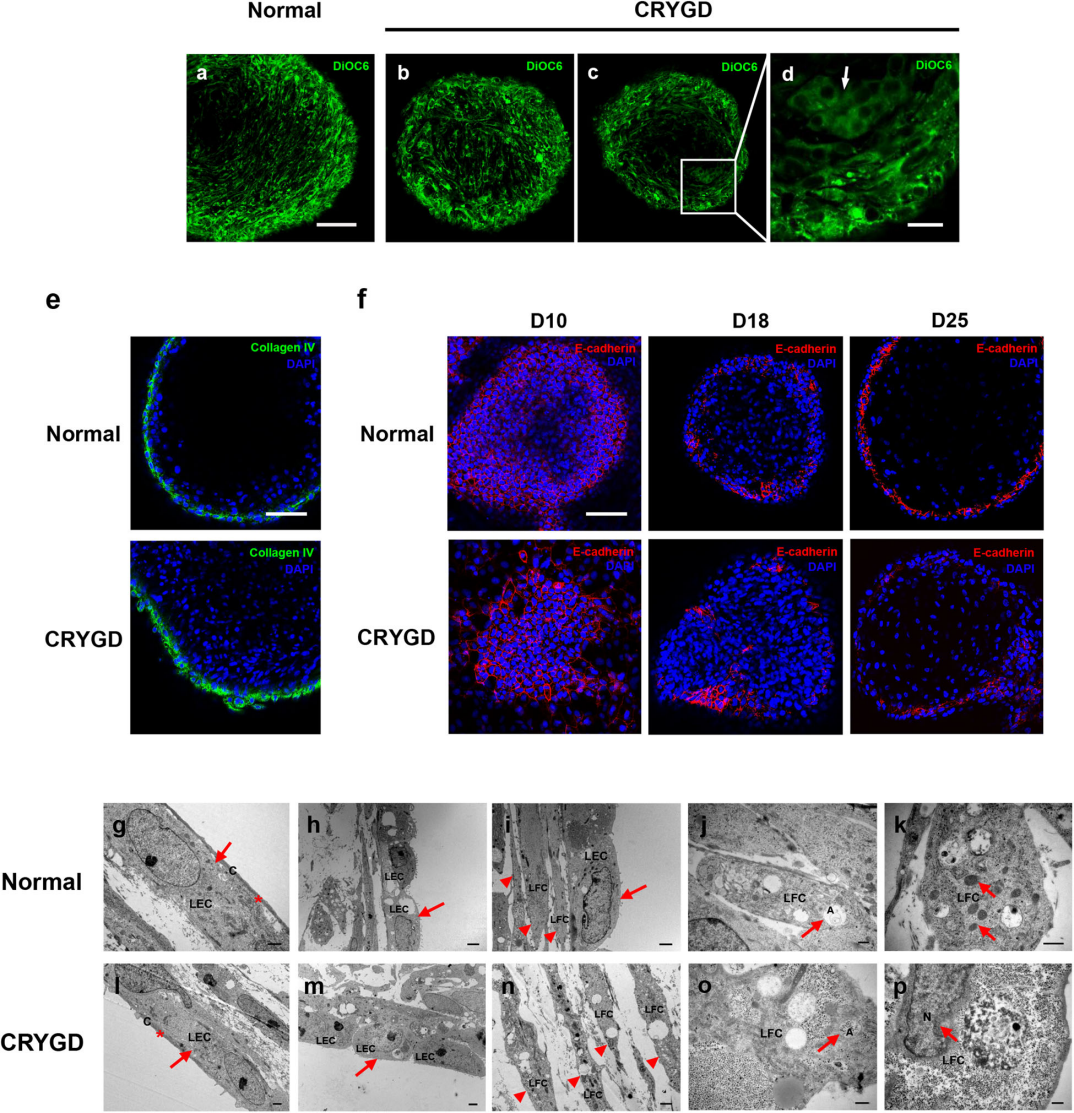
Structural analysis of the normal and the *CRYGD*-mutated LBs. **a–d** DiOC6 staining of the normal (**a**) and *CRYGD*-mutated LBs (**b–d**) on D25. The white arrow indicates cord-like structures in the *CRYGD*-mutated LBs. Scale bar: (**a–c**) 100 μm, (**d**) 25 μm. **e** Immunofluorescence analysis of collagen IV in the normal and *CRYGD*-mutated LBs on D25. Scale bar: 100 μm. **f** Immunofluorescence analysis of the lens epithelial marker E-cadherin in the normal and *CRYGD*-mutated LBs on D10, D18, and D25. Scale bar: 100 μm. **g–p** TEM images of the normal and *CRYGD*-mutated LBs on D25. Lens capsules (asterisks in **g** and **l**), lens epithelial cells (arrows in **g**, **h**, **i**, **l**, and **m**), and differentiating lens fiber cells (**j**, **k**, **o**, and **p**, arrow heads in **i** and **n**) with degenerating nuclei (arrow in **p**), degenerating organelles (arrow in **k**), and autophagosomes (arrows in **j** and **o**). Scale bars: (**g**, **l**, and **n**) 1 μm, (**h**, **i**, and **m**) 2 μm, (**j**, **k**, **o**, and **p**) 0.5 μm.

Lens-structure disorganization is considered an important feature of cataracts. Unlike normal lenses, where fibers are uniform, with a well-organized structure and organization, cataractous lenses are usually characterized by fiber cell disorder and irregularities^26,27^. To determine whether the causative mutation affected the structure of the LBs, a structural analysis of the mature *CRYGD*-mutated LBs on D25 was performed. Staining with 3,3′-dihexyloxacarbocyanine iodide (DiOC6), a membrane stain that shows the cellular arrangement, revealed that most cells in the normal LBs were regularly and closely arranged (Fig. 4a), whereas the cells in the *CRYGD*-mutated LBs were irregularly arranged, with some cord-like structures (Fig. 4b–d), which is the cellular basis of opacity in LBs. The outermost layer of collagen IV-positive cells was identified as the lens capsule, which was present in both the normal and the *CRYGD*-mutated LBs (Fig. 4e). The expression of E-cadherin, a lens epithelial marker, was detected by an immunofluorescence assay. As shown in Fig. 4f, in both the *CRYGD*-mutated and normal LBs, E-cadherin-positive cells were located diffusely in the whole-cell cluster on D10, became limited to the outer layers of the LBs on D18, and were limited to the outermost monolayer on the surface of the LBs on D25. These findings are consistent with the distribution of lens epithelial cells during embryonic lens development. However, the distribution of the E-cadherin-positive cells was more irregular in the *CRYGD*-mutated LBs than in the normal LBs.

Transmission electron microscopy (TEM) revealed a single layer of lens epithelial cells (Fig. 4g–i, l, and m) with a rectangle morphology, as well as regular nuclei and organelles, covered by a thin capsule (Fig. 4g, l). Lens fiber cells with various degrees of nuclei and organelle degeneration were observed in both the normal and the *CRYGD*-mutated LBs, indicating different stages of fiber differentiation (Fig. 4i–k, n, o, and p). Autophagosomes with a double-membrane structure were observed in some lens fiber cells (Fig. 4j and o), suggesting a process of maturation, as autophagy is thought to be involved in the degradation of nuclei and organelles during lens fiber cell differentiation.

### Patient-specific LBs showed normal differentiation during the placodal stage

**Fig. 5:**
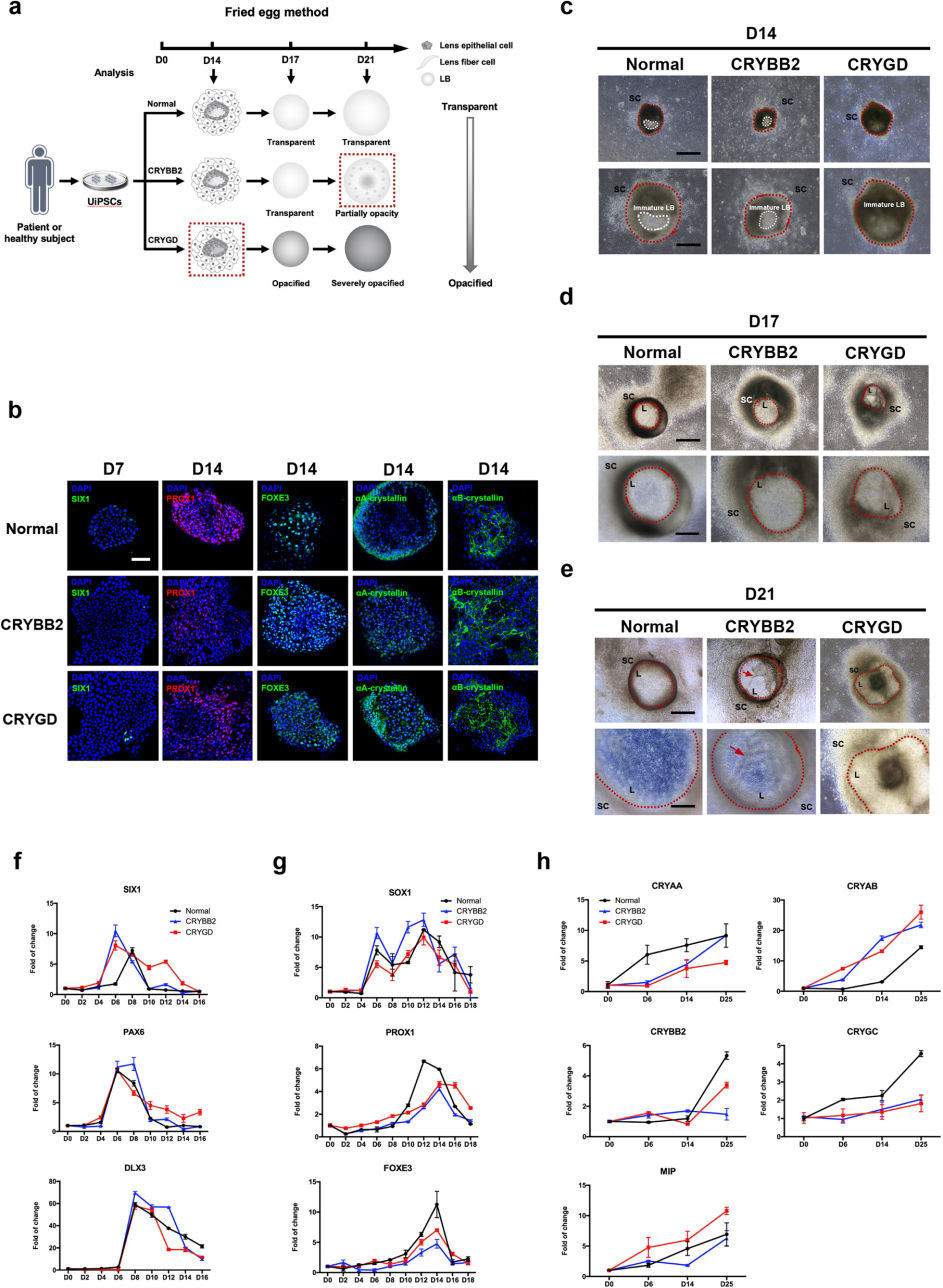
Comparative analysis of the differentiation process of the normal, *CRYGD*-, and *CRYBB2*-mutated LBs. **a** Schematic of alteration in the morphology of the normal, *CRYGD*-, and *CRYBB2*-mutated LBs during the mid-to-late stage of differentiation. **b** Immunofluorescence analysis of the placodal marker SIX1 (D7) as well as the early lens-specific markers PROX1, FOXE3, *α*A-, and αB-crystallin (D14) in the normal and patient-specific LBs. Scale bars: 100 μm. **c–e** Representative images of the normal and patient-specific LBs on D14 (**c**), D17 (**d**), and D21 (**e**), respectively. The red dashed lines indicate the borders of the LBs. The white dashed lines indicate the transparent region in the immature LBs on D14. The red arrows indicate a cord-like structure in the *CRYBB2*-mutated LBs on D21. L: LB; SC: supporting cells. Scale bars: 250 μm (upper panels), 100 μm (lower panels). **f–h** qRT-PCR analysis of the placodal markers (**f**), human early lens-specific markers (**g**), and human mature lens-specific markers (**h**) in the normal and patient-specific LBs during differentiation. The data are presented as the mean ± SD of experiments from at least three parallel samples per group.

To determine the correspondence between the progression of opacification in the patient-specific LBs and the patients’ disease courses, the different stages of LB differentiation were analyzed (schematized in Supplementary Fig. 2a and Fig. 5a). We first focused on the placodal stage during LB differentiation. The results revealed a similar process of differentiation during the placodal stage (before D8) in both the normal and the patient-specific UiPSCs (Supplementary Fig. 2b and c). As shown in Supplementary Figure 2c, colonies with a fried egg-like morphology first appeared on approximately D8 in the normal, *CRYGD*-, and *CRYBB2*-mutated groups. In terms of structure, the fried egg-like colonies were characterized by differentiating cells with a compact arrangement in the center of the colonies and supporting cells loosely arranged at the periphery. Immunofluorescence staining showed SIX1-positive cells in the colonies on D7 in the normal and the mutated groups (Fig. 5b, compared with D5 in Supplementary Fig. 2d). The qRT-PCR revealed that the expression of the placodal markers, *SIX1*, *PAX6*, and *DLX3*, peaked around D6–8 in the *CRYGD*- and *CRYBB2*-mutated LBs, which was similar to that observed in the normal LBs (Fig. 5f). These results are in accordance with that observed in patients with the *CRYGD* mutation (p. P24T) and *CRYBB2* mutation (p. Q155X), where patients usually show normal early ocular development and rarely have abnormalities in other ocular tissues, such as the retina and cornea^28^.

### Opacification occurred earlier in *the CRYGD*-mutated LBs than in the *CRYBB2*-mutated LBs during lens progenitor cell differentiation

The morphological and expression characteristics of the normal and the patient-specific LBs were compared during ongoing differentiation of lens progenitor cells (schematized in Fig. 5a). As cell differentiation progressed, the differentiating cells accumulated in the center of the colonies, with these cells having a more compact arrangement than the cells located at the periphery (Supplementary Fig. 2e). On approximately D14, immature LBs with three-dimensional structures and partial transparency first appeared at the center of the colonies (Fig. 5c). Compared with the normal LBs and *CRYBB2*-mutated LBs, the transparent area of most *CRYGD*-mutated LBs was much more limited or even absent (Fig. 5c). Considering the difference in the progression between the two patients, these findings indicated that the differentiation processes in these patient-specific LBs were similar to the disease courses of congenital cataracts caused by the corresponding mutations, respectively.

The immunofluorescence assay revealed *PROX1*- and *FOXE3*-positive cells on D14, indicating differentiation of lens progenitor cells (Fig. 5b, compared with D6 in Supplementary Fig. 2f). In addition, αA- and αB-crystallin were expressed in the *CRYGD*- and *CRYBB2*-mutated LBs as early as D14, which was similar to the observations in the normal LBs (Fig. 5b, compared with D8 in Supplementary Fig. 2g). The results of qRT-PCR revealed that the expression of the specific early lens markers, including *SOX1*, *PROX1*, and *FOXE3*, peaked around D12–14 in both the normal and patient-specific LBs (Fig. 5g).

### Opacification of patient-specific LBs was aggravated during late-stage differentiation

The late stage of differentiation of LBs was also analyzed (schematized in Fig. 5a). Compared with the normal LBs, which had a transparent and round morphology, opacification and irregular shapes were observed in the *CRYGD*-mutated LBs from D17, and opacification aggravated as differentiation progressed (Fig. 5d and e). The appearance of the *CRYBB2*-mutated LBs was relatively normal on D17, with opacification developing by around D21 (Fig. 5d and e). The expression of the lens fiber cell markers *α*A-, *α*B-, *β*-, *γ*-crystallin, and MIP throughout the process of LB differentiation, was examined. According to the results of qRT-PCR, the expressions of these markers were gradually upregulated as differentiation progressed, with expression peaking on D25. Nevertheless, *γ*-crystallin showed limited upregulation throughout the whole process of differentiation in both the *CRYGD*- and *CRYBB2*-mutated LBs (approximately two-fold of D0 on D25), as shown in Fig. 5h. Compared with the normal LBs, the *CRYGD*- and *CRYBB2*-mutated LBs displayed higher folds of upregulation of αB-crystallin during differentiation. Similar results were found in the immunofluorescence assay as mentioned above (Fig. 3a).

### Lanosterol alleviated opacifications in patient-specific LBs

**Fig. 6:**
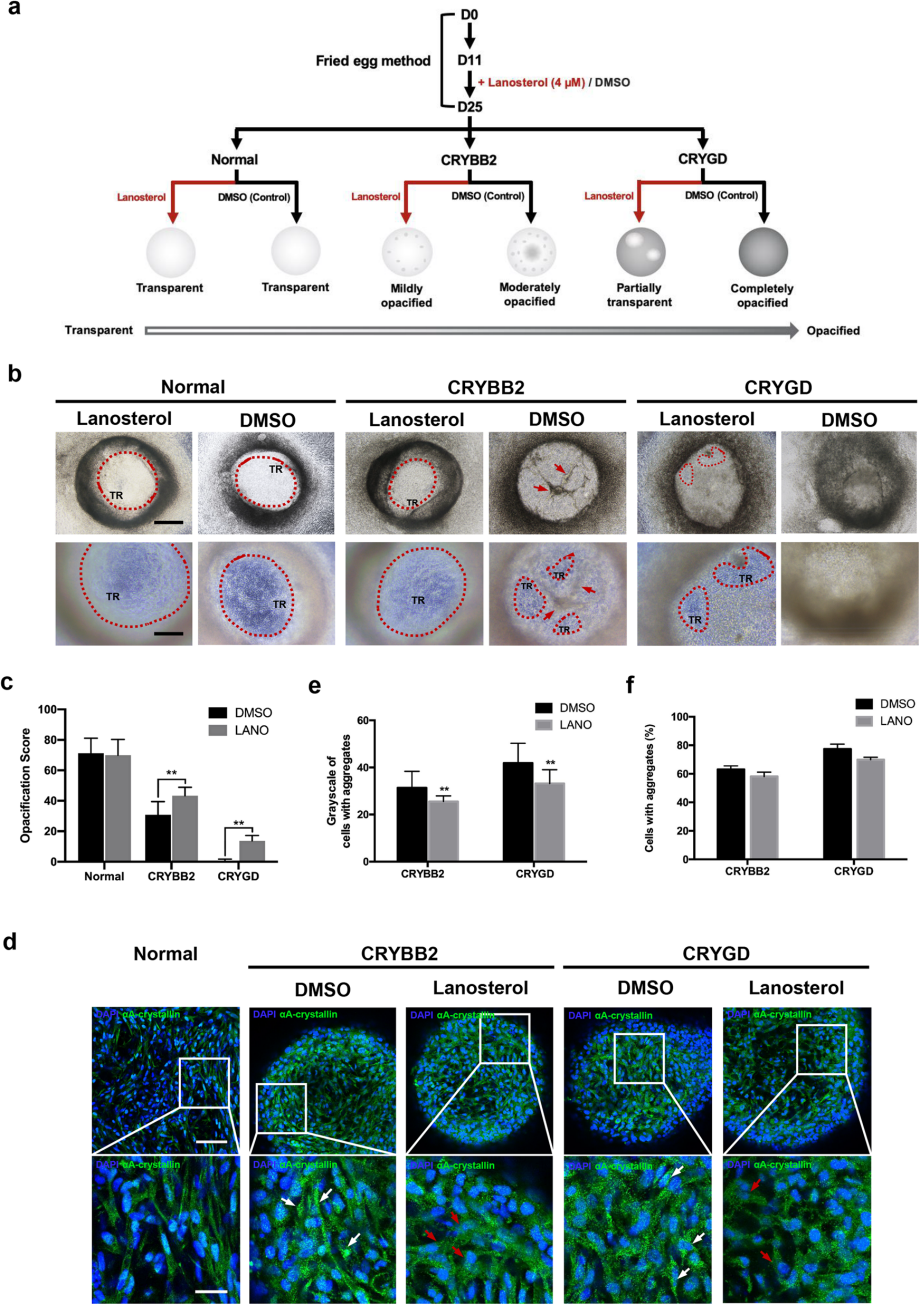
Influence of lanosterol treatment on transparency and protein aggregation in the *CRYGD*- and *CRYBB2*-mutated LBs. **a** Schematic showing morphological differences among the normal, *CRYGD*-, and *CRYBB2*-mutated LBs after lanosterol treatment. **b** Representative images of *CRYGD*- and *CRYBB2*-mutated LBs treated with lanosterol (4 μM) on D25. DMSO: treated with DMSO (1:1000 in medium) as a control. TR: transparent region. The red dashed lines indicate transparent regions. The red arrows indicate cord-like opacity in the *CRYBB2*-mutated LBs. Scale bars: 250 μm (upper panels), 100 μm (lower panels). **c** TR% analysis of the normal and patient-specific LBs treated with lanosterol or DMSO. LANO: treated with 4 μM of lanosterol. **p < 0.01 LANO versus DMSO. **d** Representative images of the αA-crystallin aggregates in the patient-specific LBs treated with lanosterol or DMSO on D25. Scale bars: 100 μm (upper panels), 25 μm (lower panels). **e**, **f** Quantitative analysis of the mean grayscales of the cells with aggregates (**e**) and the percentages of cells with protein aggregates (**f**) in the patient-specific LBs treated with lanosterol or DMSO. LANO: treated with 4 μM of lanosterol. At least three fields (250×) from each group were randomly chosen for analysis. The data are presented as mean ± SD of experiments from at least three parallel samples per group. **p < 0.01 LANO versus DMSO.

According to previous studies, lanosterol restores lens transparency in cataracts by reversing protein aggregation^24,29^. In the present study, lanosterol was administered to further confirm the similarity between the patient-specific LBs and congenital cataracts. After the continuous administration of 4 μM of lanosterol from D11 (schematized in Fig. 6a), both the *CRYGD*- and *CRYBB2*-mutated LBs revealed greater transparency than LBs treated with DMSO as control groups on D25 (Fig. 6b). The TR% values in both the *CRYGD*- and *CRYBB2*-mutated LBs were greater than those in the control groups, indicating alleviation of opacification in the patient-specific LBs (Fig. 6c). As shown in Fig. 6d, the results of the immunofluorescence assay suggested that *α*A-crystallin aggregates were decreased in the *CRYGD*- and *CRYBB2*-mutated LBs treated with lanosterol compared with the control groups. Quantitative analyses confirmed that the mean grayscale of the cells with aggregates decreased in the lanosterol-treated LBs compared with the control groups (Fig. 6e; *p* < 0.01). In addition, there was a slight decrease in the percentage of the cells with aggregates (Fig. 6f; *p* > 0.05). These findings confirmed that the patient-specific LBs showed a similar response to lanosterol to that observed in animal models of cataracts. Given the presence of a human lens-like structure, particularly an intact capsule, in LBs, they provide an ideal human model for drug-candidate screening.

## Discussion

In the present study, opacification, possibly caused by protein aggregation, was observed in the *CRYGD*-mutated LBs (p. P24T) and *CRYBB2*-mutated LBs (p. Q155X) generated from patient-specific UiPSCs based on the “fried egg” method. The patient-specific LBs shared similar characteristics in terms of clinical manifestations and disease courses to those seen in patients with cataracts, as well as similar pathological changes, providing a robust patient-specific in vitro human congenital cataract model that closely resembles cataracts in patients. This study lays the foundation for further investigations into the pathological mechanisms of congenital cataracts in vitro and the screening of drug candidates to treat cataracts.

The present study demonstrated that the morphologies of the *CRYGD*- and *CRYBB2*-mutated LBs corresponded to patients’ clinical manifestations morphologically, temporally, and molecularly. Previous studies have established models of various diseases, such as cardiac disease, Alzheimer’s disease, hepatic disease, and diabetes mellitus, based on patient-specific iPSCs that recapitulated patients’ phenotypes^1–5^. Moretti et al. modeled long-QT syndrome using patient-specific iPSCs and observed a characteristically prolonged duration of action potential in the single cells^4^. In a study by Lan et al., iPSC-derived cardiomyocytes from patients with familial hypertrophic cardiomyopathy exhibited specific pathological alterations, including cellular enlargement, multinucleation, and electrophysical and contractile arrhythmia^3^. In a patient-specific cell model of Alzheimer’s disease established by Chang et al., accumulation of Aβ, tau phosphorylation, and impairment of neurite outgrowth were observed in neurons differentiated from the patient-derived iPSCs carrying the APP D678H mutation^2^. Correspondence between the characteristics of a disease model and the disease phenotype is a key criterion of an appropriate disease model. In the present study, a patient-specific congenital cataract-disease model, which highly recapitulated patients’ lens opacification, was established, thus laying the foundation for future research into the pathological mechanisms and the screening of drug candidates to treat cataracts.

In the present study, the *CRYGD*- and *CRYBB2*-mutated LBs showed dramatically lower transparency than the normal LBs, which were similar to the patients’ cataracts. In addition, corresponding to the patients’ clinical manifestations, the opacity of the *CRYGD*-mutated LBs was more severe than that of the *CRYBB2*-mutated LBs. Previous research reported that the solubility of the P24T mutant protein in *CRYGD* was significantly lower than that of the native protein, although the protein exhibited no significant structural changes^30^. This finding may be explained by increased surface hydrophobicity in the N-terminal domain, as well as decreased backbone flexibility^30–32^. In other studies, the formation of the 4th Greek key motif was affected in the Q155X mutation in the *CRYBB2* protein, leading to altered folding properties, less normal protein–protein interactions, and increased hydrophobic interactions, thus promoting aggregation and insolubilization^33,34^. The aforementioned factors may explain why opacification occurred at an earlier stage in the *CRYGD*-mutated LBs than in the *CRYBB2*-mutated LBs in the present study. However, as the trends in *γ*-crystallin expression in the *CRYGD*- and *CRYBB2*-mutated LBs during differentiation were basically the same (Fig. 5h), the difference in opacification onset is more likely due to the different severity of the mutations. The molecular mechanisms underlying congenital cataracts will be investigated in more detail in the future using the patient-specific in vitro disease model described herein.

In the present study, the severity and type of opacification in the patient-specific LBs and patients’ cataracts were relatively similar. Interestingly, although the morphologies of the LBs derived from the same patient were roughly similar, there were some differences, for example, in terms of the distribution of the transparent regions. According to previous studies, patients with either the *CRYGD* (p. P24T) or *CRYBB2* (p. Q155X) mutation, even those from the same family, may display various phenotypes^35,36^. Therefore, it seems that in addition to the genetic background, the microenvironment of lens development may play an important role in phenotypic heterogeneity of congenital cataracts. In our previous work, we also found that the differentiation of LBs was influenced by cell density, cell-cluster size, as well as the ratio of differentiating cells and supporting cells, etc.^13^. Therefore, the slight morphological heterogeneity of the LBs in the present study may be attributed to the factors mentioned above, as well as the different microenvironment.

In the present study, the differentiation process of the patient-specific LBs represented the patient’s disease course to some degree. There was no significant distinction in the early stage of the differentiation process between the normal and patient-specific immature LBs (Supplementary Fig. 2b, c, and Fig. 5b, f). Consistently, the mutations in the *CRYGD* and *CRYBB2* genes did not affect the development of other ocular structures, including the retina and cornea^28^. Our results revealed dramatic opacity in the *CRYGD*-mutated LBs when lens progenitor cells began to differentiate into lens fiber cells (Fig. 5c). Such opacity is due to the synthesis of γD-crystallin, which is synthesized mainly in lens fiber cells during the embryonic stage^36^. This accounts for the obvious opacification of LBs during the maturation of lens fiber cells and explains the leukocoria in proband 1 since birth. According to previous researches, some causative gene mutations would result in a deficiency in lens fiber differentiation^37^, which may also account for the decreased expression of γ-crystallin in the *CRYGD*- and *CRYBB2*-mutated LBs in the present study. To elucidate the mechanism underlying cataractogenesis, it is important to determine the time point when a cataract appears, which is not possible in a human fetus. Congenital cataract murine models may be an alternative, but the species difference remains an important limitation. Therefore, patient-specific LBs may provide an ideal model to shed light on the disease course in cataractogenesis.

In the present study, expressional and structural changes were present in the patient-specific LBs compared with the normal LBs. The results of the qRT-PCR and immunofluorescence assays revealed lower expression of *γ*-crystallin in both the *CRYGD*- and *CRYBB2*-mutated LBs than in the normal LBs (Figs. 3a, b and 5h). Similar results have been reported elsewhere, with research showing that a mutation in the *Hsf4* gene could lead to reduced *γ*-crystallin expression, indicating defects in secondary fiber differentiation^37–40^. Therefore, mutations in the *CRYGD* and *CRYBB2* genes may also have adverse effects on the differentiation of lens fiber cells. Of note, in the present study, the expressions of *α*B-crystallin were higher in the *CRYGD*- and *CRYBB2*-mutated LBs than in the normal LBs (Figs. 3b and 5h). *α*B-crystallin is a small heat-shock protein, which not only prevents protein aggregation but also protects lens cells from stresses, such as UV irradiation, oxidation, and chemical denaturants^41^. Herein, we put forward the hypothesis that the upregulation of *α*B-crystallin may be an intrinsic compensatory mechanism to protect lens cells from adverse effects caused by mutations. As shown in Fig. 4b–d, the *CRYGD*-mutated LBs were characterized by a disorganized cellular arrangement as compared with the normal LBs. This result supports the lack of normal fiber organization in cataractous lenses, as reported by previous studies^26,27,40^. The present study demonstrated that lanosterol partially prevented the development of opacification and protein aggregates in the *CRYGD*- and *CRYBB2*-mutated LBs. Although there was only a slight decrease in the percentage of the cells with aggregates in the patient-specific LBs treated with lanosterol, the results of the grayscale measurements indicated that lanosterol significantly reduced intracellular protein aggregation (Fig. 6d–f). The results of the blank control are not presented, as there was no distinct difference in either LB morphology or aggregate formation. Zhao et al. reported that lanosterol significantly reduced intracellular aggregation of mutant crystallin proteins, with an efficacy of up to 60% in cells expressing βB2-crystallin (V187E) or *γ*D-crystallin (W43R) mutants^24^. Compared with these results, the efficacy of lanosterol in the present study was lower. Differences in the mutant crystallin proteins, as well as the treatment concentrations of lanosterol, may partly explain this finding. Previous research also reported that the efficacy of lanosterol may be dependent upon cataract severity^29^. Thus, differences in cataract severity may explain the different efficacies of lanosterol in the patient-specific LBs with different mutations. Moreover, it is worth mentioning that in LBs, the capsule may confine the permeation of lanosterol, thereby greatly reducing the drug concentration in the LBs and thus the efficacy. Therefore, with the presence of intact capsules, LBs better simulate the human lens structure and provide an ideal and unique human platform for anticataract drug screening and evaluation.

Cellular and animal models provide a good platform for the study of pathological mechanisms and anticataract drug evaluation. Human cellular models expressing various causative genes enable functional research of specific mutations^42–44^, and animal models enable the disease process to be followed morphologically, histologically, and biochemically^45–48^. Based on these models, the mechanisms of congenital cataracts have been demonstrated to a large degree, and the efficacy of several drug candidates, including lanosterol and 25-hydroxycholesterol, in treating cataracts, has been demonstrated^23,24^. However, these models also have limitations. On the one hand, two-dimensional human cellular models are not fully representative of the human lens in vivo in terms of morphological, structural, and biochemical characteristics. On the other hand, the application of animal models is confined by various factors, including the heterogeneity of the genetic background, long experimental periods, difficulty in efficiently acquiring point mutations, and high costs. Patient-specific LBs well overcome the shortcomings of both cellular and animal models. First, patient-specific LBs provide a feasible and convenient method to develop human congenital cataract models with various mutations, including point mutations. Second, LBs only take about 10 days of drug treatment, whereas animals usually require repetitive intravitreous injections or drop administration for weeks or even months. Third, as previously mentioned, the structure of LBs closely resembles that of the human lens, especially the outermost capsule. Therefore, patient-specific LBs may serve as an ideal alternative disease model of congenital cataracts and contribute to optimizing the system of mechanism, as well as therapeutic study. In the future, this patient-specific lens-regeneration technology may be combined with gene-editing technologies, such as CRISPR/Cas9, to correct cataract-causing mutations in vitro and be applied in the regenerative therapy of congenital cataracts.

The present study has some limitations. Although the patient-specific LBs closely mimicked the patients’ clinical manifestations, only two causative mutations of congenital cataracts were studied, and only one family with each mutation was included. In a future study, a comprehensive system of patient-specific congenital cataract models will be established by generating LBs from patients with different causative mutations, as well as patients with the same mutation and different phenotypes. This system will also be included in screening and evaluation of drug candidates to treat cataracts.

In summary, the present study established a patient-specific congenital cataract model in vitro via induced differentiation of patient-specific UiPSCs into LBs. The patient-specific LBs shared similarities with the patients’ cataracts in terms of their morphologies, disease courses, and pathological changes. Therefore, the present study provided a promising platform for the research on pathological mechanisms and clinical drug-candidate screening for congenital cataracts in the future.

## Methods

### Collection of the clinical data and donor samples

The study protocol adhered to the tenets of the Declaration of Helsinki and was approved by the Medical Ethics Committee of the Second Affiliated Hospital of Zhejiang University School of Medicine, Hangzhou, China. Two families with *a CRYGD* mutation (p. P24T) and the *CRYBB2* mutation (p. Q155X), respectively, were recruited. Slit-lamp photographs of eyes with dilated pupils were obtained from the electronic medical record system, with the participants’ consent. After written informed consent from the participants was obtained, periphery blood samples and urine samples were collected from the proband and two unaffected family members from each family.

### Generation and identification of UiPSCs

Exfoliated urinary cells from the urine samples were induced into iPSCs according to the method described by Zhou et al. and ourselves in a previous study^13,49^. Briefly, the urinary cells were infected with a combination of retroviruses encoding the human transcription factors SOX2, OCT-4, KLF4, and c-MYC. The infected cells were then seeded onto mouse embryonic fibroblasts treated with mitomycin C (Cell Bank, Shanghai, China). Two-to-three weeks later, the UiPSC colonies were picked mechanically and cultured in mTeSR^TM^ 1 medium (StemCell Technologies, Vancouver, CA, USA) on plates coated with Matrigel (Corning, NY, USA).

### AP staining

The UiPSCs were fixed with 4% paraformaldehyde (PFA) in a phosphate buffer (PBS, 0.1 M, pH 7.0) and then incubated in an AP staining solution (mix Fast Red Violet [FRV]) with Napthol AS-BI phosphate solution and water in a 2:1:1 ratio (FRV:Naphthol:water) by using the Alkaline Phosphatase Detection Kit (Millipore, Billerica, USA) according to the manufacturer’s protocol. Finally, the images were captured using an Olympus IX71 microscope (Olympus) equipped with DP2-BSW software (Olympus).

### Teratoma assay for UiPSCs

NOD/SCID mice (6–8 weeks old, male) were purchased from Silaike Experimental Animal Co. (Shanghai, China). All animal experiments were approved by the Institutional Animal Care and Use Committee at Zhejiang University. Mice were housed in a standard environment with a temperature of 20–24 °C and a humidity of 50–60% under a 12-h light-dark cycle with food and water provided ad libitum. In total, 1 × 10^6^ UiPSCs were injected into the muscle center in the hind-leg quadriceps along the long axis of the mice. Mice were observed for tumor formation every day. Then mice were sacrificed with intraperitoneal injection of an overdose of 2% pentobarbital sodium, usually at 6–8 weeks after injection of UiPSCs. The tumors were isolated, fixed in formalin, and embedded in paraffin. The specimens were then sectioned into 5-μm slices using a RM2245 Microtome (Lecia). Hematoxylin–eosin staining was performed and the images of sections were then captured using a DM 4000B microscope (Leica).

### Mutation analysis

Genomic DNA samples from the affected and unaffected family members were isolated from the peripheral blood samples using QIAamp DNA Blood Kits (QIAGEN, Hilden, Germany). Genomic DNA samples were also extracted from the UiPSCs derived from all the subjects using the Wizard SV Genomic DNA Purification System (Promega, Madison, WI, USA). Exon 2 of the *CRYGD* gene and exon 6 of the *CRYBB2* gene were amplified with PCR and the primer sequences are listed in Supplementary Table 1. After agarose gel electrophoresis, the PCR products were purified using a DNA Gel Extraction Kit (AxyPrep, San Francisco, CA, USA). Sanger sequencing was performed (Sangon, Shanghai, China), and the results were analyzed using SnapGene software (v4.1.8, SL Biotech, Chicago, IL, USA). The primers used for Sanger sequencing are listed in Supplementary Table 1.

### Differentiation of LBs derived from UiPSCs

LBs were differentiated from UiPSCs as described in detail in our previous study^13^. In brief, the cells were treated with mTeSR^TM^ 1 medium supplemented with 100 ng/mL of Noggin (R&D, Minneapolis, MN, USA) for six days, followed by mechanical isolation and reseeding of the desired cell clusters into new dishes. Noggin was then replaced by 20 ng/mL of BMP4 (R&D), 20 ng/mL of BMP7 (R&D), and 100 ng/mL of bFGF (PeproTech, Rocky Hill, NJ, USA) for the following nine days. Typical fried egg-like structures appeared around D11 of differentiation. Finally, BMP4 and BMP7 were then replaced by 20 ng/mL of Wnt3a (R&D) to complete the process of differentiation. Mature LBs were obtained around D25. Images of the LBs were captured at various time points using an Olympus IX71 microscope (Olympus, Tokyo, Japan) equipped with DP2-BSW software (v2.2, Olympus).

### qRT-PCR assay

Total RNA was extracted from the cells during LB differentiation on D0, D2, D4, D6, D8, D10, D12, D14, D16, D18, D21, D23, and D25 using a QIAGEN RNeasy Mini Kit (QIAGEN) according to the manufacturer’s guidelines. Cell clusters that did not form LB structures were discarded to avoid influence on the results. The quality and concentration of the RNA samples were detected using a NanoDrop 2000c spectrophotometer (Thermo Fisher Scientific, Waltham, MA, USA). The cDNA was synthetized through reverse transcription. To quantify the expression of specific markers of lens differentiation, a qRT-PCR assay was performed using TB Green^®^ *Premix Ex Taq*^™^ (TaKaRa, Beijing, China) on an ABI Fast 7500 RT-PCR system (v2.0.6, Life Technologies, Carlsbad, CA, USA), according to the manufacturer’s protocols. The details of the data processing are described in our previous study^13^. All the primers used for qRT-PCR are listed in Supplementary Table 1.

### Immunofluorescence assay

The LBs at various time points during differentiation and the UiPSCs were fixed with 4% paraformaldehyde (PFA) in a phosphate buffer (PBS, 0.1 M, pH 7.0), permeabilized with 0.5% Triton X-100 (Sigma-Aldrich) in PBS for 25 min, and then incubated overnight with primary antibodies, followed by incubation with secondary antibodies. Nuclei were labeled with 1 µg/mL of 4,6-diamido-2-phenylindole dihydrochloride (DAPI) (Sigma-Aldrich). Images were captured using a Leica TCS SP8 confocal microscope (Leica, Wetzlar, Germany) or an Olympus IX71 microscope (Olympus) and processed with Image J software (v1.0, National Institutes of Health [NIH], DC, USA). The antibodies and dilutions used are listed in Supplementary Table 2. Cells with aggregates were counted using Image J software (NIH). At least three fields (250×) from each group were randomly chosen for analysis.

### Quantification of opacification

Images of the mature LBs on D25 were captured using an Olympus IX71 microscope (Olympus). The TR% within each of the LBs was measured using Image J software (NIH), as illustrated in Fig. 2a. Three ophthalmologists blinded to the study protocol independently measured the TR% within each of the LBs.

### DiOC6 staining

On D25, the mature LBs were fixed with 4% PFA in PBS, permeabilized with 0.5% Triton X-100 (Sigma-Aldrich) in PBS for 25 min, and then incubated overnight with 5 mg/mL of DiOC6 (Sigma-Aldrich). Images were captured using a Leica TCS SP8 confocal microscope (Leica) at 488 nm and processed with Image J software (NIH).

### Soluble and insoluble protein extraction and analysis

Cell samples of mature LBs on D25 were collected and lysed with a lysis buffer containing phenylmethanesulfonyl fluoride (PMSF), protease inhibitor, and phosphatase inhibitors (Sangon). Half of the total protein samples were centrifuged at 16,000 rpm for 30 min at 4 °C. The supernatant was collected as the soluble proportion, and the precipitate was resuspended in PBS with an equal amount of the supernatant and collected as the insoluble proportion. The total, soluble, and insoluble protein were stored at −80 °C. A Pierce BCA Protein Assay Kit (Thermo Fisher Scientific) was used for protein quantification. The protein concentration was determined using an iMark Microplate Absorbance Reader spectrophotometer (Bio-Rad, Hercules, CA, USA). The protein extracts from each sample were loaded into an SDS-PAGE gel and dyed with Coomassie Blue Super-Fast Staining Solution (Beyotime, Shanghai, China). The gels were washed overnight and the bands of proteins were detected using a Bio-Rad ChemiDoc MP Imaging System (Bio-Rad).

### TEM analysis

The LBs were harvested on D25 and fixed with 2.5% glutaraldehyde in PBS overnight, followed by postfixation with 1% OsO_4_ in PBS for 2 h. The samples were dehydrated in a graded series of ethanol, and they were then infiltrated and embedded in Spurr’s resin. Finally, using a Leica EM UC7 Ultratome (Leica), LB-specimen sections 70–90-nm thick were obtained and stained with uranyl acetate and alkaline lead citrate for 5 and 10 min, respectively. The sectioned LBs were observed using a Hitachi Model H-7650 TEM (Hitachi, Tokyo, Japan).

### Optic property analysis of LBs (“X” assay)

The optic properties of the LBs were analyzed as previously described^13^. Briefly, 35-mm culture dishes with the LBs were placed on a paper printed with an “X”. A dissecting microscope (Olympus) was then used to observe the “X” through the LBs. The images of the “X” under an empty dish or through the LBs were taken using a Pro-MicroScan digital camera (Olympus) equipped with ToupView software (v1.1, ToupTek, Hangzhou, China).

### Lanosterol treatment

Lanosterol powder (Sigma-Aldrich, St. Louis, MO, USA) was dissolved in dimethyl sulfoxide (DMSO) (Sigma-Aldrich) at a concentration of 4 mM and stored at −80 °C. Then, 4 μM of lanosterol (1:1000) was continuously administered to the *CRYGD*- and *CRYBB2*-mutated UiPSC-derived cells from around D11 when an intact fried egg-like structure appeared. The cells treated with DMSO (1:1000) were used as the control group.

### Statistical analysis

The data are presented as the mean ± standard deviation (SD) of experiments from at least three parallel samples per group. A one-way analysis of variance followed by a post hoc multiple comparison using the least-significant-difference test was performed using IBM SPSS Statistics software (v25.0.0.0, IBM, Armonk, NY, USA) for comparison of more than two groups that was required. Two-sided *p*-values less than 0.05 were considered significant.

### Reporting summary

Further information on research design is available in the Nature Research Reporting Summary linked to this article.

## Supplementary information

Supplementary Information

Reporting Summary

## Data Availability

The authors declare that the main data supporting the findings of this study are available within the article and its Supplementary Information files. Extra data are available from the corresponding author upon reasonable request.
