# Supplementary material for: Modeling congenital cataract in vitro using patient-specific induced pluripotent stem cells
Source: NPJ Regen Med. 2021 Oct 1;6:60. doi: 10.1038/s41536-021-00171-x (PMC8486789; doi:10.1038/s41536-021-00171-x)
Supplement: Supplementary file 1 — Supplementary Information [file 41536_2021_171_MOESM1_ESM.pdf]

Supplementary Information

Supplementary Figure 1. Characterization of the UiPSCs derived from proband 1 and proband 2.

Supplementary Figure 2. Comparative analysis of the differentiation process of the normal, *CRYGD*-, and *CRYBB2*-mutated lentoid bodies (LBs) during the early-middle stage of differentiation.

Supplementary Table 1. Primer sequences.

Supplementary Table 2. Antibodies used for immunofluorescence assay.

Supplementary Figure 1

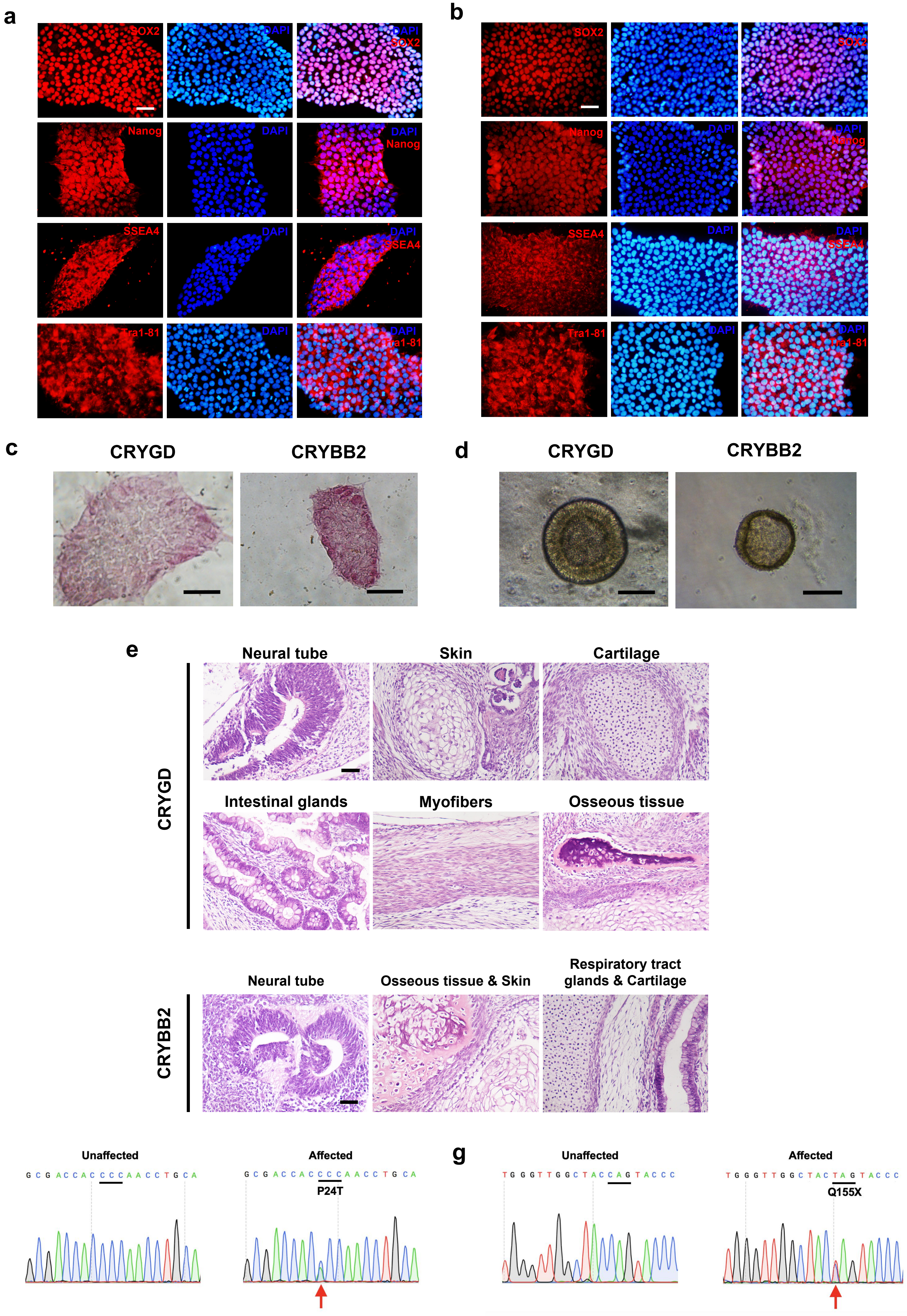

Supplementary Figure 1. Characterization of the UiPSCs derived from proband 1 and proband 2. (a & b) Immunofluorescence analysis of embryonic stem-cell antigens Nanog, SSEA4, SOX2, and TRA1-81 in the UiPSCs derived from proband 1 (a) and proband 2 (b). Scale bar: 50 μm. (c) Alkaline phosphatase (AP) staining of the UiPSCs derived from proband 1 (left panel) and proband 2 (right panel). Scale bar: 25 μm. (d) Embryonic body formation assay of the UiPSCs derived from proband 1 (left panel) and proband 2 (right panel). Scale bar: left panel, 100 μm; right panel, 250 μm. (e) H&E staining of the teratomas formed from the UiPSCs derived from proband 1 and 2 in the NOD/SCID mice. Scale bar: 50 μm. (f & g) Sanger sequencing analysis of the UiPSCs derived from the affected and unaffected individuals in family 1 (f) and family 2 (g).

Supplementary Figure 2

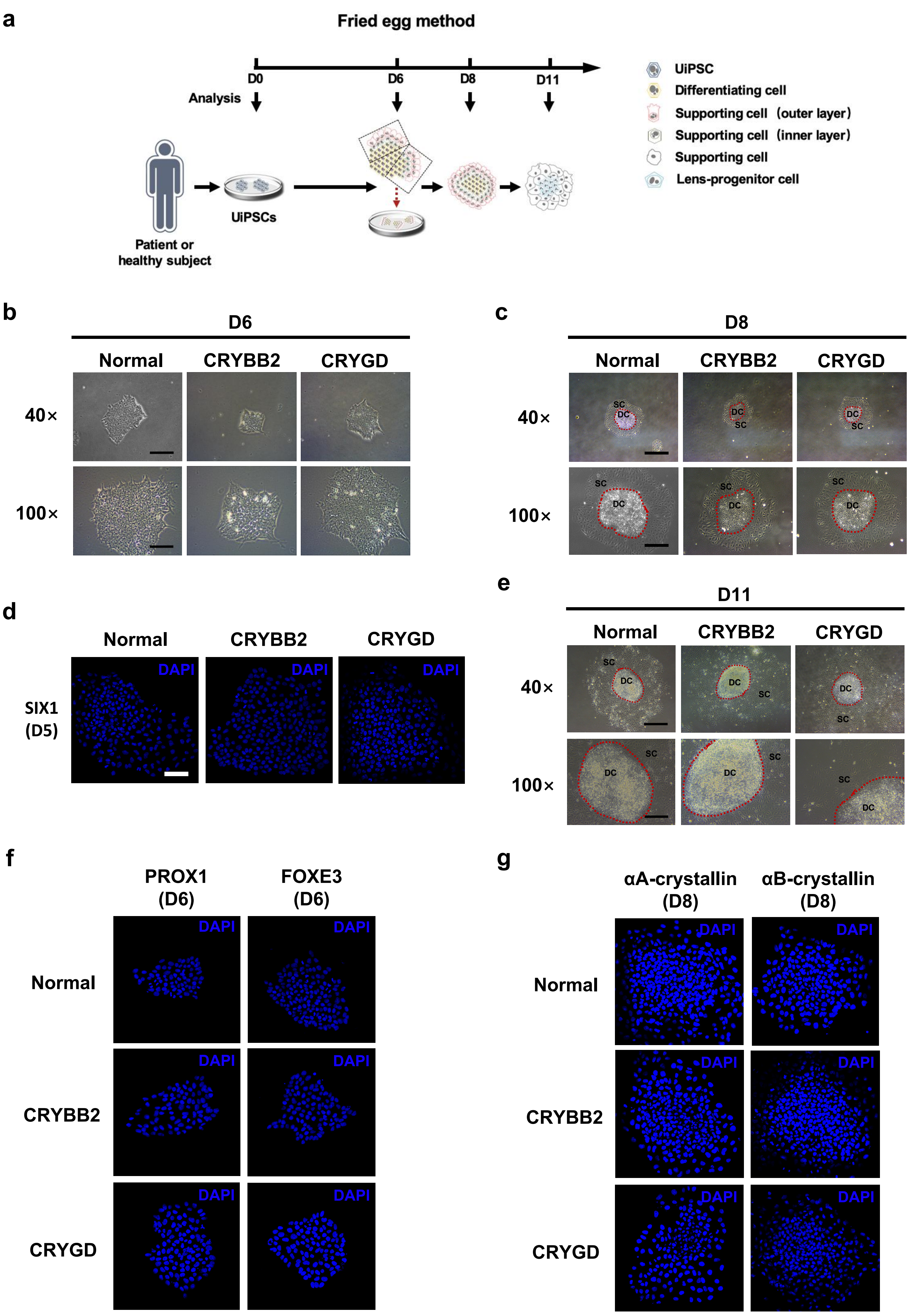

Supplementary Figure 2. Comparative analysis of the differentiation process of the normal, *CRYGD*-, and *CRYBB2*-mutated lentoid bodies (LBs) during the early-middle stage of differentiation. (a) Schematic of the morphology alteration of the normal, *CRYGD*-, and *CRYBB2*-mutated LBs during the early-middle stage of differentiation. (b & c) Representative images of the normal and patient-specific mutated LBs on D6 (b) and D8 (c). DC: differentiating cells. SC: supporting cells. The dashed lines indicate the borders between differentiating cells and supporting cells. Scale bars: 250 μm (upper panels), 100 μm (lower panels). (d) Immunofluorescence analysis of the placodal marker SIX1 in the normal, the *CRYGD*- and *CRYBB2*-mutated LBs on D5. Scale bars: 100 μm. (e) Representative images of the normal and patient-specific mutated LBs on D11. DC: differentiating cells. SC: supporting cells. The red dashed lines indicate the borders between differentiating cells and supporting cells. Scale bars: 250 μm (upper panels), 100 μm (lower panels). (f) Immunofluorescence analysis of the early lens specific markers PROX1 and FOXE3 in the normal, the *CRYGD*- and *CRYBB2*-mutated LBs on D6. Scale bars: 100 μm. (g) Immunofluorescence analysis of the early lens specific markers αA- and αB-crystallin in the normal, the *CRYGD*- and *CRYBB2*-mutated LBs on D8. Scale bars: 100 μm.

Supplementary Table 1. Primer sequences.

Name	Direction	Primer sequence (5'→3')
**Exon amplification**		
CRYBB2 Exon-6	Forward	AGTGGCAATGGTTGGGAGG
	Reverse	AGTCACACTTTATTCACTCTCTGGGAG
CRYGD Exon-2	Forward	AGCTTCCTCCATCGCGG
	Reverse	CATCCAGTGAGTGTCCTGAGGG
**Sanger sequencing**		
CRYBB2		AGTGGCAATGGTTGGGAGG
CRYGD		AGCTTCCTCCATCGCGG
**Quantitative real-time PCR**		
CRYAA	Forward	AAGGTGCAGGACGACTTTGT
	Reverse	GTGGAACTCACGGGAAATGT
CRYAB	Forward	GTTCTTCGGAGAGCACCTGTT
	Reverse	GAGAGTCCAGTGTCAAACCAG
CRYBB2	Forward	GCAGGTTCTGTCCTAGTGCAG
	Reverse	CTCTTGGCTGTCCACTTTGAT
CRYGC	Forward	TGAGCGTCCCAACTACCAAG
	Reverse	GTGGAGGGAACGGATCTCG
MIP	Forward	GTGCTGTATAGCGTTACCCCA
	Reverse	CTCGTCGTATGTGGCAAAGAT
PROX1	Forward	AAAGTCAAATGTACTCCGCAAGC
	Reverse	CTGGGAAATTATGGTTGCTCCT
SOX1	Forward	GCAGGTCCAAGCACTTACAAG
	Reverse	TATAACTCCGCCGTCTGAAGG
FOXE3	Forward	GCCTCCAGTGAGTCCATA
	Reverse	AATCTCCAAGAAGTGTCCTC

DLX3	Forward	CTCGCCCAAGTCGGAATATAC
	Reverse	CTGGTAGCTGGAGTAGATCGT
SIX1	Forward	CTGCCGTCGTTTGGCTTTAC
	Reverse	GCTCTCGTTCTTGTGCAGGT
PAX6	Forward	TTTGCCCGAGAAAGACTAGC
	Reverse	CATTTGGCCCTTCGATTAGA
GADPH	Forward	ATTGCCCTCAACGACCACT
	Reverse	ATGAGGTCCACCACCCTGT

Supplementary Table 2. Antibodies used for immunofluorescence assay.

Antibody	Brand	Cat No.	Source	Dilution
**Primary Antibodies**				
αA-Crystallin polyclonal antibody	ENZO	ADI-SPA-221	Rabbit	1:100
αB-Crystallin monoclonal antibody (1B6.1-3G4)	ENZO	ADI-SPA-222	Mouse	1:100
β-Crystallin antibody	Santa Cruz	sc-22745	Rabbit	1:100
γ-Crystallin antibody	Santa Cruz	sc-22746	Rabbit	1:100
MIP (AQP40) antibody	Santa Cruz	sc-99059	Rabbit	1:100
SIX1 (D4A8K) Rabbit mAb	Cell Signaling	12891	Rabbit	1:100
E-Cadherin (24E10) Rabbit mAb	Cell Signaling	3195	Rabbit	1:100
Anti-FOXE3 antibody produced in rabbit	Sigma-Aldrich	AV32304	Rabbit	1:100
PROX1 (D2J6J) Rabbit mAb	Abcam	14963	Rabbit	1:100
Anti-Collagen IV antibody	Abcam	Ab6586	Rabbit	1:100
Anti-SOX2 antibody	Millipore	AB5603	Rabbit	1:250

Human Nanog Antibody	R&D	AF1997	Goat	1:75
Anti-SSEA4 antibody [MC813]	Abcam	ab16287	Mouse	1:75
Anti-TRA1-81 Antibody, clone TRA-1-81, Cy3 conjugate	Millipore	MAB4381C3	Mouse	1:100
**Secondary Antibodies**				
Anti-rabbit IgG (H+L), F(ab')$_2$ Fragment (Alexa Fluor®555 Conjugate)	Cell Signaling	4413	Goat	1:1000
Anti-mouse IgG (H+L), F(ab')$_2$ Fragment (Alexa Fluor®555 Conjugate)	Cell Signaling	4409	Goat	1:1000
Anti-rabbit IgG (H+L), F(ab')$_2$ Fragment (Alexa Fluor®488 Conjugate)	Cell Signaling	4412	Goat	1:1000
Anti-mouse IgG (H+L), F(ab')$_2$ Fragment (Alexa Fluor®488 Conjugate)	Cell Signaling	4408	Goat	1:1000
Donkey anti-Goat IgG (H+L) Cross-Adsorbed Secondary Antibody, Alexa Fluor 555	Invitrogen	A-21432	Donkey	1:1000
