# Supplementary material for: Modeling congenital cataract in vitro using patient-specific induced pluripotent stem cells
Source: NPJ Regen Med. 2021 Oct 1;6:60. doi: 10.1038/s41536-021-00171-x (PMC8486789; doi:10.1038/s41536-021-00171-x)
Supplement: Supplementary file 2 — Reporting Summary [file 41536_2021_171_MOESM2_ESM.pdf]

nature portfolio

Corresponding author(s): Ke Yao, Qiuli Fu

Last updated by author(s): Jun 25, 2021

Reporting Summary

Nature Portfolio wishes to improve the reproducibility of the work that we publish. This form provides structure for consistency and transparency in reporting. For further information on Nature Portfolio policies, see our Editorial Policies and the Editorial Policy Checklist.

Please do not complete any field with "not applicable" or n/a. Refer to the help text for what text to use if an item is not relevant to your study. For final submission: please carefully check your responses for accuracy; you will not be able to make changes later.

Statistics

For all statistical analyses, confirm that the following items are present in the figure legend, table legend, main text, or Methods section.

n/a	Confirmed	
☐	☒	The exact sample size (*n*) for each experimental group/condition, given as a discrete number and unit of measurement
☐	☒	A statement on whether measurements were taken from distinct samples or whether the same sample was measured repeatedly
☐	☒	The statistical test(s) used AND whether they are one- or two-sided<br>*Only common tests should be described solely by name; describe more complex techniques in the Methods section.*
☒	☐	A description of all covariates tested
☒	☐	A description of any assumptions or corrections, such as tests of normality and adjustment for multiple comparisons
☒	☐	A full description of the statistical parameters including central tendency (e.g. means) or other basic estimates (e.g. regression coefficient) AND variation (e.g. standard deviation) or associated estimates of uncertainty (e.g. confidence intervals)
☒	☐	For null hypothesis testing, the test statistic (e.g. *F*, *t*, *r*) with confidence intervals, effect sizes, degrees of freedom and *P* value noted<br>*Give P values as exact values whenever suitable.*
☒	☐	For Bayesian analysis, information on the choice of priors and Markov chain Monte Carlo settings
☒	☐	For hierarchical and complex designs, identification of the appropriate level for tests and full reporting of outcomes
☒	☐	Estimates of effect sizes (e.g. Cohen's *d*, Pearson's *r*), indicating how they were calculated

Our web collection on statistics for biologists contains articles on many of the points above.

Software and code

Policy information about availability of computer code

Data collection	Data of qRT-PCR were acquired on an ABI Fast 7500 RT-PCR system v2.0.6 (Life Technologies, Carlsbad, CA, USA).
Data analysis	The results of Sanger sequencing were analyzed using SnapGene software v4.1.8 (SL Biotech, Chicago, IL, USA).<br>Quantitative analyses of IF images, LB opacification, and gels were performed on Image J software v1.0 (National Institutes of Health [NIH], DC, USA).<br>Statistical analyses were performed using IBM SPSS Statistics software version v25.0.0.0 (IBM, Armonk, NY, USA).

For manuscripts utilizing custom algorithms or software that are central to the research but not yet described in published literature, software must be made available to editors and reviewers. We strongly encourage code deposition in a community repository (e.g. GitHub). See the Nature Portfolio guidelines for submitting code & software for further information.

Data

Policy information about availability of data

All manuscripts must include a data availability statement. This statement should provide the following information, where applicable:

- Accession codes, unique identifiers, or web links for publicly available datasets
- A description of any restrictions on data availability
- For clinical datasets or third party data, please ensure that the statement adheres to our policy

The authors declare that the main data supporting the findings of this study are available within the article and its Supplementary Information files. Extra data are available from the corresponding author upon reasonable request.

Field-specific reporting

Please select the one below that is the best fit for your research. If you are not sure, read the appropriate sections before making your selection.

☒ Life sciences ☐ Behavioural & social sciences ☐ Ecological, evolutionary & environmental sciences

Life sciences study design

All studies must disclose on these points even when the disclosure is negative.

Sample size	The data are acquired from experiments from at least three parallel samples per group. Though no sample-size calculation was performed, replication from at least three parallel samples is acknowledged as adequate.
Data exclusions	No data were excluded.
Replication	The reproducibility of data has been confirmed by replication of at least three parallel samples per group.
Randomization	This is not relevant to the present study since the experimental and control groups were determined according to the iPSCs derived from patients and healthy individuals, respectively.
Blinding	The LB opacification quantification was performed by three ophthalmologists independently using a blind method. Blinding was not relevant to other assays since there is no potential bias.

Reporting for specific materials, systems and methods

We require information from authors about some types of materials, experimental systems and methods used in many studies. Here, indicate whether each material, system or method listed is relevant to your study. If you are not sure if a list item applies to your research, read the appropriate section before selecting a response.

Materials & experimental systems

n/a	Involved in the study	
☐	☒	Antibodies
☒	☐	Eukaryotic cell lines
☒	☐	Palaeontology and archaeology
☐	☒	Animals and other organisms
☐	☒	Human research participants
☒	☐	Clinical data
☒	☐	Dual use research of concern

Methods

n/a	Involved in the study	
☒	☐	ChIP-seq
☒	☐	Flow cytometry
☒	☐	MRI-based neuroimaging

Antibodies

Antibodies used

αA-Crystallin polyclonal antibody ENZO ADI-SPA-221
αB-Crystallin monoclonal antibody (1B6.1-3G4) ENZO ADI-SPA-222
β-Crystallin antibody Santa Cruz sc-22745
γ-Crystallin antibody Santa Cruz sc-22746
MIP (AQP40) antibody Santa Cruz sc-99059
SIX1 (D4A8K) Rabbit mAb Cell Signaling 12891
E-Cadherin (24E10) Rabbit mAb Cell Signaling 3195
Anti-FOXE3 antibody produced in rabbit Sigma-Aldrich AV32304
PROX1 (D2J6J) Rabbit mAb Abcam 14963
Anti-Collagen IV antibody Abcam Ab6586
Anti-SOX2 antibody Millipore AB5603
Human Nanog Antibody R&D AF1997
Anti-SSEA4 antibody [MC813] Abcam ab16287
Anti-TRA1-81 Antibody, clone TRA-1-81, Cy3 conjugate Millipore MAB4381C3
Anti-rabbit IgG (H+L), F(ab')2 Fragment (Alexa Fluor®555 Conjugate) Cell Signaling 4413
Anti-mouse IgG (H+L), F(ab')2 Fragment (Alexa Fluor®555 Conjugate) Cell Signaling 4409
Anti-rabbit IgG (H+L), F(ab')2 Fragment (Alexa Fluor®488 Conjugate) Cell Signaling 4412
Anti-mouse IgG (H+L), F(ab')2 Fragment (Alexa Fluor®488 Conjugate) Cell Signaling 4408
Donkey anti-Goat IgG (H+L) Cross-Adsorbed Secondary Antibody, Alexa Fluor 555 Invitrogen A-21432

Validation

All primary antibodies have been validated.

Animals and other organisms

Policy information about studies involving animals; ARRIVE guidelines recommended for reporting animal research

Laboratory animals	NOD/SCID mice
Wild animals	The study did not involve wild animals.
Field-collected samples	The mice were housed in a standard environment with a temperature of 20 to 24°C and a humidity of 50 to 60% under a 12-hour light-dark cycle with food and water provided ad libitum. UiPSCs were injected into the muscle center in the hind-leg quadriceps along the long axis of the mice. Animals were sacrificed with intraperitoneal injection of an overdose of 2% pentobarbital sodium for isolation of tumors , usually at 6-8 weeks after the injection.
Ethics oversight	All animal experiments were approved by the Institutional Animal Care and Use Committee at Zhejiang University.

Note that full information on the approval of the study protocol must also be provided in the manuscript.

Human research participants

Policy information about studies involving human research participants

Population characteristics	Congenital cataract patients and healthy individuals without urinary tract diseases or other ocular diseases were recruited.
Recruitment	Subjects were recruited in outpatient department. No potential bias is involved.
Ethics oversight	The study protocol was approved by the Medical Ethics Committee of the Second Affiliated Hospital of Zhejiang University School of Medicine, Hangzhou, China.

Note that full information on the approval of the study protocol must also be provided in the manuscript.

This checklist template is licensed under a Creative Commons Attribution 4.0 International License, which permits use, sharing, adaptation, distribution and reproduction in any medium or format, as long as you give appropriate credit to the original author(s) and the source, provide a link to the Creative Commons license, and indicate if changes were made. The images or other third party material in this article are included in the article's Creative Commons license, unless indicated otherwise in a credit line to the material. If material is not included in the article's Creative Commons license and your intended use is not permitted by statutory regulation or exceeds the permitted use, you will need to obtain permission directly from the copyright holder. To view a copy of this license, visit http://creativecommons.org/licenses/by/4.0/
